# Telemedicine Adoption for Managing Chronic and Rare Diseases in Indonesia During and Beyond the COVID-19 Era: Qualitative Study

**DOI:** 10.2196/83462

**Published:** 2026-03-19

**Authors:** Christine Gracia Pratama, Rima Nurlianti, Cornelius Herstatt, Moritz Goeldner

**Affiliations:** 1 Technology and Innovation Management, Hamburg University of Technology (TUHH), Am Schwarzenberg-Campus 4, Hamburg, 21073, Germany, 49 40 30601 4777; 2 Leuphana University of Lüneburg, Lüneburg, Germany; 3 University of Auckland, Auckland, New Zealand; 4 Working Group for Data-Driven Innovation, Hamburg University of Technology, Hamburg, Germany

**Keywords:** digital health, telemedicine, chronic disease, rare disease, COVID-19, Indonesia

## Abstract

**Background:**

Telemedicine has emerged as a valuable tool for improving health care delivery, especially in low-resource and geographically isolated regions. In Indonesia, the COVID-19 pandemic highlighted the need for digital health solutions to manage chronic diseases and improve access to specialists.

**Objective:**

This study aimed to examine the rapid adoption of telemedicine in Indonesia, focusing on its role in managing chronic and rare diseases and highlighting both its benefits and challenges to long-term viability during and post pandemic.

**Methods:**

A qualitative study was used, following the Consolidated Criteria for Reporting Qualitative Research 32-item checklist and guided by established theories and frameworks. Recruitment followed a systematic multistage procedure suited to the context of Indonesia, an upper-middle-income country and Group of Twenty member characterized by wide regional and socioeconomic disparities. Data were collected through 15 semistructured interviews conducted between October and December 2024. The interviews were divided into 2 phases: first with physicians from various medical specialties and 6 out of 7 regions within Indonesia, and then with patients diagnosed with autoimmune diseases. All interviews were audio-recorded, transcribed verbatim, and analyzed inductively to identify recurrent themes and subthemes.

**Results:**

Before the COVID-19 pandemic, telemedicine adoption in Indonesia was limited, with many physicians recalling its nonexistence before 2015. The pandemic accelerated the adoption of virtual platforms, which became essential for patient consultation, follow-ups, and medication management. Though its use decreased after the pandemic, telemedicine remains valuable, particularly for chronic and rare disease management in remote areas with limited access to specialists. At least three overarching themes were identified: (1) acceleration and normalization of telemedicine (the pandemic catalyzed the integration of virtual consultations into routine care, transforming initial skepticism into broad acceptance); (2) accessibility and cost efficiency (telemedicine reduced financial and travel burdens, especially for patients in remote areas, with several reporting cost savings from hybrid consultation models); and (3) limitations of virtual care (physicians highlighted constraints related to the absence of physical examination).

**Conclusions:**

The findings reveal that telemedicine has become a valuable tool in Indonesia for managing chronic and rare diseases, particularly for follow-up care and medical specialist access across diverse geographical areas. While telemedicine has improved health care accessibility and demonstrated significant cost benefits by reducing transportation and consultation costs, challenges such as limited infrastructure and physician burnout remain. Long-term success will depend on the development of sustainable regulatory frameworks, continued investment in digital infrastructure, and a focus on optimizing cost-effectiveness in health care delivery.

## Introduction

### Background

Digital technologies offer new ways to improve health care coverage and quality, particularly in settings with limited resources and widespread geographic dispersion. Integrating digital technologies has made care more efficient, accessible, and patient-centered. The global adoption of digital health care, such as telemedicine, was accelerated by the COVID-19 pandemic [1-3]. Telemedicine has gained prominence as a crucial component of health care delivery. It was strongly advocated during the pandemic for its potential to address immediate health care needs while reducing the risk associated with in-person visits [4-6]. This shift was not just a temporary solution; it marked the beginning of a permanent transformation in health care delivery models in both the Global North and the Global South. Importantly, telemedicine also holds potential to support progress toward sustainable development goal (SDG) 3 (good health and well-being) and goal 10 (reducing inequalities) by enabling more equitable access to health care services across diverse socioeconomic and geographic contexts.

With a population exceeding 270 million and more than 17,500 islands, Indonesia faces significant disparities in health care access due to its fast-growing population, urbanization, and huge distances between health care providers and patients. This intensified during the pandemic, particularly among individuals with chronic, noncommunicable diseases (NCDs) [7]. Therefore, digital health solutions have the potential to ensure equitable and continuous care in this region. Early evidence suggests that telemedicine could reduce transportation costs by 4% and shorten patient travel times without compromising the level of care [8]. However, amidst the effectiveness of telemedicine, barriers such as digital literacy, educational level, age, rural residence, and internet access continue to affect its adoption [9-11].

### Evolution, Definitions, and Current Relevance

The digital transformation of health care systems has the potential to greatly improve quality while reducing costs [12,13]. Telemedicine, which originated in the early 20th century with the use of telephone lines to transmit medical data, has evolved significantly and become a vital component of modern health care delivery. Early applications, such as remotely transmitting electrocardiograms and X-rays, laid the groundwork for telemedicine’s role in improving health care access, particularly in emergencies and rural areas [14].

The World Health Organization defines telemedicine as the use of information and communication technology to provide health care remotely, particularly in regions with limited access to medical services [12]. Telemedicine is highly patient-centered because it is convenient and caters to patients’ digital preferences. This makes telemedicine an essential component of patient care management in areas with limited traditional options [15]. Telemedicine enables communication between health care providers and patients through technology [16]. It encompasses both synchronous and asynchronous methods of delivering health care services remotely and is provided through various digital platforms, including secure messaging, mobile apps, and video conferencing [16-19]. Telemedicine is a broad health care service that uses telecommunication and electronic technologies to support remote consultations, monitoring, nursing, and rehabilitation between patients and health care providers [4,20].

### Telemedicine Adoption in Asia-Pacific and Organization for Economic Cooperation and Development Countries

In the Asia-Pacific region, telemedicine experienced substantial growth during the COVID-19 pandemic, nearly doubling between 2019 and 2021. Australia recorded a ninefold increase in usage during this period [20,21]. However, postpandemic trends show a stabilization or decline in some countries. For instance, between 2021 and 2023, Singapore and Australia each saw a 9 percentage points rise in telemedicine adoption (from 34% to 43% and 45% to 54%, respectively), while Indonesia’s adoption increased by 7 percentage points (from 51% to 58%) [22,23]. Conversely, from the same study, India experienced a 4 percentage points decline (from 59% to 55%), reflecting regional disparities in digital infrastructure, regulatory support, and user engagement.

In Organization for Economic Cooperation and Development countries, telemedicine use was limited before the pandemic, with an average of only 0.6 teleconsultations per patient annually in 2019 [24]. Sweden led in adoption, with 47% of Swedish adults reporting in early 2021 that they had at least 1 teleconsultation since the start of the pandemic [25,26]. France also saw a dramatic spike, from 136,882 consultations in 2019 to 4.5 million in April 2020 alone, driven by policy changes such as full reimbursement [27]. Germany’s growth was short-lived; after a surge from 3000 consultations in 2019 to 2.7 million in 2020, usage declined by 40% in 2022 [28,29]. Physician adoption mirrored this trend: in Sweden, 50% of physicians conducted 25%‐75% of consultations online weekly, compared to just 6% in France and 14 % in Germany [30]. These patterns underscore the need for adaptive digital health policies tailored to each country’s health care context [31].

### Digital Health Adoption and COVID-19

The COVID-19 pandemic catalyzed an unprecedented acceleration in digital adoption across health care systems globally, particularly in the area of telemedicine. Mobile apps offering teleconsultation, contact tracing, symptom monitoring, and remote patient management became essential tools for maintaining health care continuity during lockdowns. Regional distinctions shaped the design and use of these platforms: for example, Asian countries emphasized user accessibility and functional simplicity to promote widespread adoption, while North American and European apps primarily focused on disseminating health information with limited interactivity [32]. Additionally, global differences in regulatory environments, such as Germany’s stringent data privacy laws compared to India’s more rapid, utilitarian deployment of contact-tracing apps, reflect diverse policy priorities during the pandemic [33]. These variances underscore the importance of localized strategies for telehealth implementation and regulation, particularly as countries transition into the postpandemic phase.

Post-COVID-19 telemedicine is increasingly recognized as a foundational element of modern health care, extending far beyond its initial role as an emergency response. The World Health Organization emphasized its potential not only for acute care but also as a sustainable solution for managing long-term health conditions. Chronic diseases, which demand ongoing monitoring and personalized care, have emerged as a central focus for telehealth apps [5,6]. This is particularly relevant for rare diseases, which often require specialist consultations and coordinated care that may not be readily available in all geographic regions. The decentralized model enabled by telemedicine can mitigate geographic and systemic barriers, facilitating access to specialist expertise for rare disease patients and improving outcomes through timely interventions and continuity of care [34,35].

Indonesia exemplifies the strategic integration of telemedicine into national health planning in the postpandemic context. With a population exceeding 270 million distributed across thousands of islands, the government has prioritized digital health to overcome logistical challenges and expand health care accessibility [36]. The Ministry of Health’s “Blueprint of Digital Transformation Strategy 2024” developed in collaboration with the United Nations Development Program outlines a comprehensive framework for digital health system development [37,38]. This includes infrastructure to support the management of chronic and rare diseases, particularly in view of the increasing prevalence of NCDs in the country [7,39]. The blueprint reflects a broader regional trend toward embedding telehealth within long-term health care policy, signaling a shift from reactive digital health adoption to proactive, system-wide transformation. Such strategies are vital to ensuring equitable care for patients with chronic and rare diseases in both urban and remote settings.

### Use of Telemedicine for Chronic and Rare Disease Management

Chronic and rare disease management requires a continuous, coordinated, and patient-centered approach, focusing on monitoring, medication adherence, education, and preventive care to improve outcomes and quality of life [40,41]. Chronic NCD diseases such as diabetes, cardiovascular conditions, and autoimmune disorders—including rare diseases like systemic lupus erythematosus and Sjögren’s disease—demand sustained health care interventions due to their complexity and long-term nature [42,43]. Telemedicine has emerged as a critical enabler of chronic care, particularly during and after the COVID-19 pandemic, by bridging geographic gaps, reducing travel costs, and supporting remote management for patients in underserved areas [32,44,45]. In Indonesia, for example, the Program Pengelolaan Penyakit Kronis under Badan Pengelola Jaminan Sosial Kesehatan (BPJS; National Health Insurance Agency) illustrates the integration of telemedicine in chronic disease management, offering education, routine monitoring, and preventive care for diabetes and hypertension [24,46].

The post-pandemic era has highlighted telemedicine’s broader clinical use in managing both chronic and rare diseases. Studies demonstrate its efficacy in improving medication adherence and reducing psychological distress among patients with conditions such as rheumatoid arthritis, while also enhancing satisfaction in postoperative and psychiatric care when delivered via video consultations [47-50]. Although many autoimmune patients still prefer initial face-to-face consultations, hybrid models—where video is used for follow-ups—have been well received, with 84% reporting video consultations as effective when paired with subsequent in-person visits [43]. Beyond synchronous video, asynchronous tools such as SMS have proven particularly effective among younger patients, promoting continuous engagement through reminders and 2-way communication [51]. To ensure quality and patient safety, frameworks like the Asia-Pacific League of Associations for Rheumatology telemedicine guidelines recommend time-limited follow-ups, prioritization through preconsultation screening, and strict adherence to data privacy standards [52]. Collectively, these strategies underscore telemedicine’s critical role in enhancing access, continuity, and personalization of care for patients with complex and chronic conditions.

### Cost-Benefit Analysis

Telemedicine has emerged as a cost-effective solution for improving health care access and quality, particularly in regions facing shortages of medical professionals. In Association of Southeast Asian Nation countries, including Indonesia, the scarcity of physicians in rural areas exacerbates health care challenges. With only 7.5 medical practitioners per 10,000 population, Indonesia lags behind other nations like Malaysia and Singapore, where the availability of physicians is much higher. Telemedicine closes this gap by offering remote consultations, which reduces the need for patients to travel and minimizes health care costs for patients and providers alike [49]. The increasing adoption of telemedicine has proven to reduce consultation, travel, and time-related expenses, while also enhancing the overall quality of care.

Studies from Europe also highlight the cost-effectiveness of telemedicine. A comprehensive analysis found that telemedicine was cost-effective in 73.3% of cases, with reduced consultation costs and travel expenses being significant contributors to savings [53]. In Sweden, for example, digital consultations cost 41.5% less than in-person visits [54]. Similarly, a remote monitoring program for diabetic patients in France demonstrated cost savings, with the remote group spending €1334 (US $1513, December 2021 exchange rate) less compared to the control group over 1 year [55]. These findings underscore the potential of telemedicine to reduce both health care costs and improve patient outcomes, particularly in systems where teleconsultations are integrated into public health systems with reimbursement options.

Telemedicine also shows considerable promise in reducing nonmedical expenses, such as transportation costs and lost time from work or school. Studies from Bangladesh and pediatric rheumatology research highlight the substantial savings telemedicine provides by cutting travel time by 56% and associated costs by 94% [43,56]. In Indonesia, the cost of teleconsultations varies depending on the platform, with independent companies offering more affordable options compared to hospital-integrated services. However, telemedicine remains largely inaccessible through public health insurance, as it is only reimbursed via private insurance or self-payment [57]. Despite these limitations, text messaging and other telemedicine tools continue to be cost-effective solutions for chronic disease management, particularly in underserved areas with limited health care access [58,59]. This qualitative study aims to examine how access to telemedicine supports care for managing chronic and rare diseases in Indonesia, drawing on perspectives from both patients and health care professionals in the post-pandemic context.

## Methods

### Ethical Considerations

Informed consent was obtained from all participants prior to data collection. Data were collected, anonymized, and stored securely in line with international ethical standards. No personal or social information was collected, and all data was fully anonymized to ensure privacy and confidentiality. Participants were not offered any compensation for taking part in the study.

This study was conducted in accordance with the principles of the Declaration of Helsinki. Ethical approval was obtained from the German Association for Experimental Economic Research Institutional Review Board (certificate number syBSVy9b). No identifying images or personal or clinical details of participants are included.

### Research Design

This study adopts a qualitative research design following the Consolidated Criteria for Reporting Qualitative Research (COREQ) 32-item checklist as provided as Checklist 1 [60,61]. The topic guides were developed by RN and CGP in accordance with the COREQ 32-item checklist to ensure comprehensive coverage of key methodological domains. Guide development was informed by prior literature on telemedicine adoption and qualitative interviewing and subsequently reviewed by MG to establish content validity. A deductive analytic approach was applied, drawing on established theories and frameworks to inform the research questions and interview guide development.

The study comprises two primary participant groups: physicians and patients. Semistructured interviews were chosen as the main data collection method due to their suitability for eliciting in-depth responses on telemedicine experiences and challenges [62,63]. The interviews were conducted in 2 phases. First, physicians from different regions and medical specialties were interviewed. Next, case studies of patients diagnosed with autoimmune diseases were examined.

### Data Collection

#### Recruitment

Recruitment followed a systematic multistage procedure suited to the context of Indonesia, an upper-middle-income country and Group of Twenty member characterized by wide regional and socioeconomic disparities [64]. For physicians, the inclusion criteria required active medical practice in Indonesia, prior experience using or familiarity with telemedicine in managing chronic or rare diseases, and willingness to participate in an online interview. For patients, inclusion criteria included having a clinically confirmed chronic or rare autoimmune condition and experience with telemedicine services. A social media-based strategy was used, as it has proven effective in engaging participants and achieving broader outreach across socioeconomic categories [65-68]. Physicians were approached through professional platforms (LinkedIn and ResearchGate), email, and Instagram, while patients were recruited through content-sharing platforms (Instagram and TikTok) and WhatsApp community groups. This multiplatform strategy enabled access to both health care professionals and hard-to-reach patient populations [66,68,69]. Previous studies underscored notable gender disparities, revealing a predominance of female patients, which corresponds with the higher incidence of autoimmune diseases observed in women [69].

Furthermore, standardized invitations outlining the study, eligibility criteria, and confidentiality were shared, thus participation occurred according to respondents’ availability. Digital consent was obtained prior to interviews. Although social media enabled broad outreach, engagement from marginalized groups remained uneven [65].

For this study, Indonesia was categorized into Western and Eastern regions. Western Indonesia comprises Sumatra, Java, and Kalimantan, while Eastern Indonesia includes Nusa Tenggara, Sulawesi, Maluku, and Papua. Participants were drawn from 6 of Indonesia’s 7 major regions, including Sumatra, Java, Kalimantan, Nusa Tenggara, Sulawesi, and Papua. Recruitment from the Maluku Islands proved challenging due to the absence of referral connections with physicians or patients with autoimmune conditions, as well as the region’s small population.

National telecommunications statistics indicate substantial regional disparities in digital connectivity. Western Indonesia generally demonstrates higher internet access, with an average of 80% of the population, compared to around 40% in the Eastern regions [70]. The lowest internet penetration was observed in parts of Papua, where access declined to approximately 6% in 2024 [70]. Similar results were found for cellular signal coverage across 7 Indonesian regions [70].

#### Interviews With Physicians and Patients

A total of 35 physicians were approached, resulting in 15 semistructured interviews conducted between August and November 2024. The participants, representing 6 medical specialties, were chosen for their experience with telemedicine, research involvement, or public health advocacy. For physicians, the inclusion criteria required active medical practice in Indonesia, prior experience using or familiarity with telemedicine in managing chronic or rare diseases, and willingness to participate in an online interview. For patients, inclusion criteria included having a clinically confirmed chronic or rare autoimmune condition and experience with telemedicine services. Each interview, conducted in Bahasa Indonesia via Zoom (Zoom Communications, Inc) or WhatsApp (Meta Platforms, Inc), lasted 25 to 63 minutes (mean 48, SD 13.4), following a 5-section interview guide with 27 questions as shown in Appendix S1 (Multimedia Appendix 1). In total, the interviews yielded 743 minutes of recordings and 163 pages of transcripts. The interviews were primarily conducted by RN, with several sessions co-led or supported by CGP. Physicians were recruited through social media, professional platforms, and referrals, with 1 session including a passive observer (a community leader).

In the second phase, 15 patients diagnosed with autoimmune diseases were contacted, leading to 9 interviews conducted between November 15 and December 15, 2024. All interviews were conducted by RN. Recruitment took place through patient communities on Instagram (Meta Platforms, Inc) and WhatsApp (Meta Platforms, Inc), with consent from community leaders. Each interview had a typical duration of 26 to 70 minutes (mean 54, SD 16.1), guided by a 6-section, 34-question format, and transcribed verbatim. Appendix S3 (Multimedia Appendix 1) contains the interview guide, and a detailed interview summary is attached in Appendix S4 (Multimedia Appendix 1). The total output was 539 minutes of recordings and 197 transcript pages, with 1 session also involving a nonparticipant community leader. Table 1 summarizes key details of both phases.

**Table 1.** Overview of interview characteristics and participant details.

Category	Physicians	Patients
Interview period	October 10-November 10, 2024	November 15-December 15, 2024
Participants reached	35 physicians	15 patients
Number of participants	15 physicians	9 patients
Recruitment method	Direct outreach via ResearchGate, LinkedIn, Instagram, and referral through mutual connections	Community posts and broadcasts on Instagram and WhatsApp
Language	Bahasa Indonesia	Bahasa Indonesia
Interview duration	25-63 min (mean 48, SD 13.4)	26-70 min (mean 54, SD 16.1)
Interview format	Semistructured (5 sections and 27 questions)	Semistructured (6 sections and 34 questions)
Total recorded time	743 min	539 min
Transcript length	163 pages	197 pages

### Data Analysis

**Figure 1.**
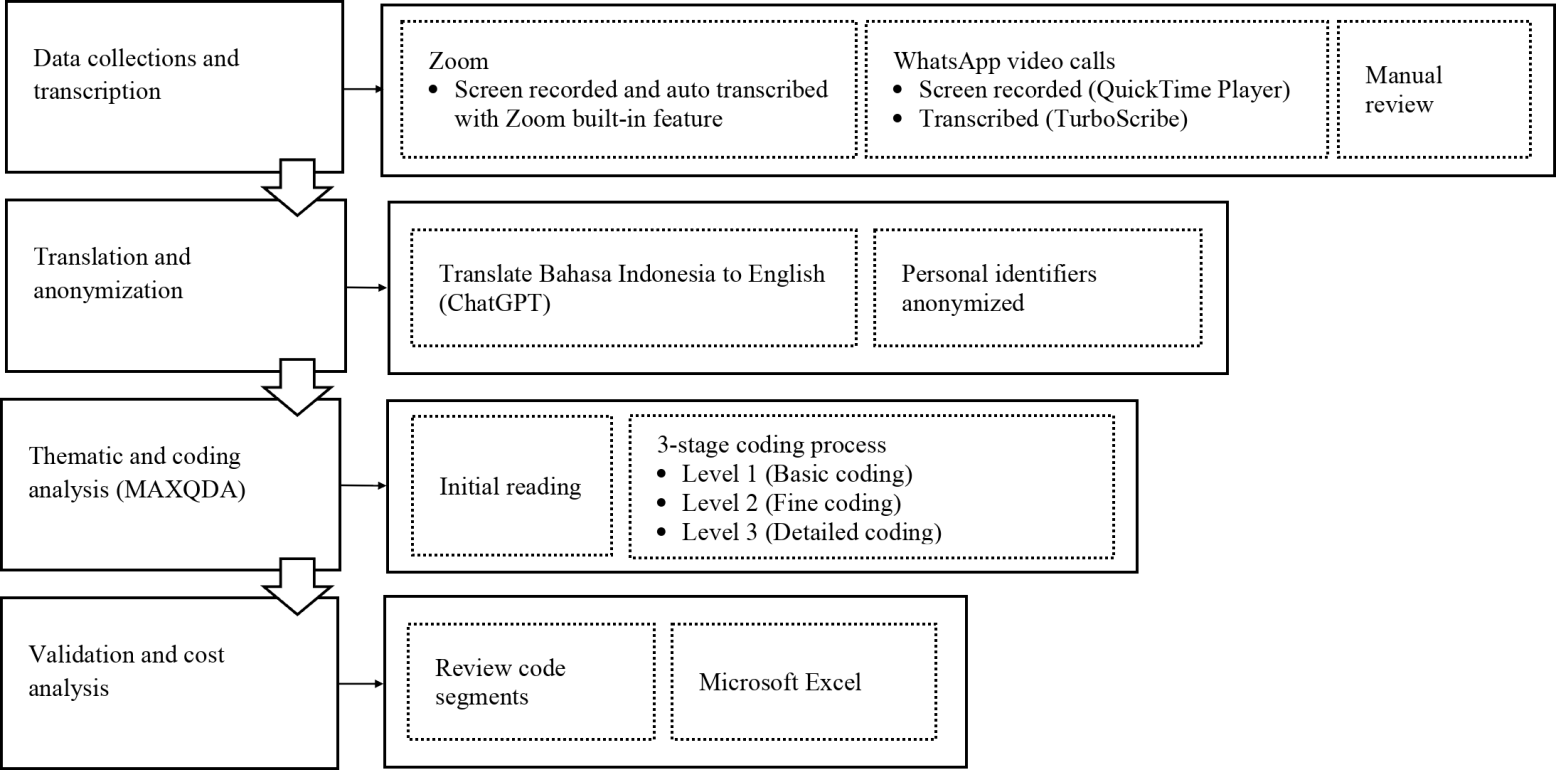
Steps involved in the qualitative data analysis process.

Thematic analysis is used to systematically identify, analyze, and interpret patterns within qualitative data [71]. Transcripts were initially reviewed for accuracy and completeness. Zoom (Zoom Communications, Inc) interviews were transcribed using built-in features, while WhatsApp (Meta Platform) recordings were processed using QuickTime Player (Apple Inc) and TurboScribe (Leif Erikson Ventures, LLC). All transcripts were manually checked and then translated into English using ChatGPT 4.0 (OpenAI) and DeepL (DeepL SE) to ensure consistency and accessibility for non-Indonesian—speaking readers. All translated transcripts were then reviewed again manually by 2 of the authors, both of whom are native speakers and 1 of whom has medical knowledge. To maintain confidentiality and reduce participant burden, transcripts were not returned to participants for verification. Identifying details were anonymized throughout. The data analysis process is displayed in Figure 1.

Data were analyzed using MAXQDA (VERBI Software GmbH), following a structured, 3-level coding process [72]. First-level coding identified broad themes that served as the foundation for analysis. Second-level coding, or fine coding, further refined these categories into specific subcategories for deeper analysis of telemedicine experiences. Third-level coding introduced additional granularity, breaking down subcategories into more specific classifications to capture nuanced patterns within the data. Coding was primarily conducted by RN, while CGP and MG independently reviewed portions of the transcripts to validate emerging codes and themes. Theme validation extended beyond partial co-coding through iterative comparison of the final themes with the original transcripts to ensure accurate representation of participants’ perspectives. Interpretive discrepancies were discussed among the authors and resolved through consensus. In addition, the researchers also held regular discussions throughout the coding process and participated in debriefing sessions with MG to further strengthen analytical rigor. Data saturation was determined by RN during the coding process and subsequently confirmed through joint discussions with CGP and MG. Saturation was considered achieved once participants from all major Indonesian regions were represented and recurring patterns consistently emerged across interviews. This determination was further supported by verification through field notes and preliminary coding reviews, with no additional first-order concepts or second-order themes identified [73].

In addition to thematic analysis, cost-related data mentioned in patient interviews (eg, transportation, consultation, and medication costs) were extracted and compiled using Microsoft Excel (Microsoft Corporation) to support an exploratory cost-benefit dimension.

Interviews with physicians were thematically coded across 6 domains: general experience, telemedicine usage, perceived benefits, challenges, health care accessibility, and future outlook. Physicians highlighted telemedicine’s effectiveness in monitoring stable patients, especially in remote areas, citing improved accessibility and cost-efficiency. However, they also noted challenges such as diagnostic limitations, poor internet connectivity, legal concerns, and disparities in patient digital literacy. Telemedicine adoption varied by region, case complexity, and health care setting, with usage patterns influenced by the COVID-19 pandemic and shifting postpandemic practices. The coding structure is visualized in Appendix S5 (Multimedia Appendix 1).

Patient interviews were classified according to demographics, medical history, telemedicine usage, health care access and costs, challenges, and overall perceptions. Participants reported diverse experiences shaped by illness severity, hospital class, payment methods (eg, BPJS and private insurance), and regional access to specialists. Many appreciated the reduced travel and financial burden telemedicine offered, while others preferred in-person care for complex diagnostics. Barriers included infrastructure limitations, insurance gaps, and varying levels of digital literacy. Overall, patients valued the flexibility of telemedicine but saw it as a complementary tool rather than a full replacement for face-to-face consultations. Cost-related findings were supported by comparative analysis using MAXQDA (VERBI Software GmbH) and Microsoft Excel (Microsoft Corporation) to highlight reductions in nonmedical expenses. Appendix S6 (Multimedia Appendix 1) presents the coding analysis level of the patient.

## Results

### Geographic Distribution of Participants

**Figure 2.**
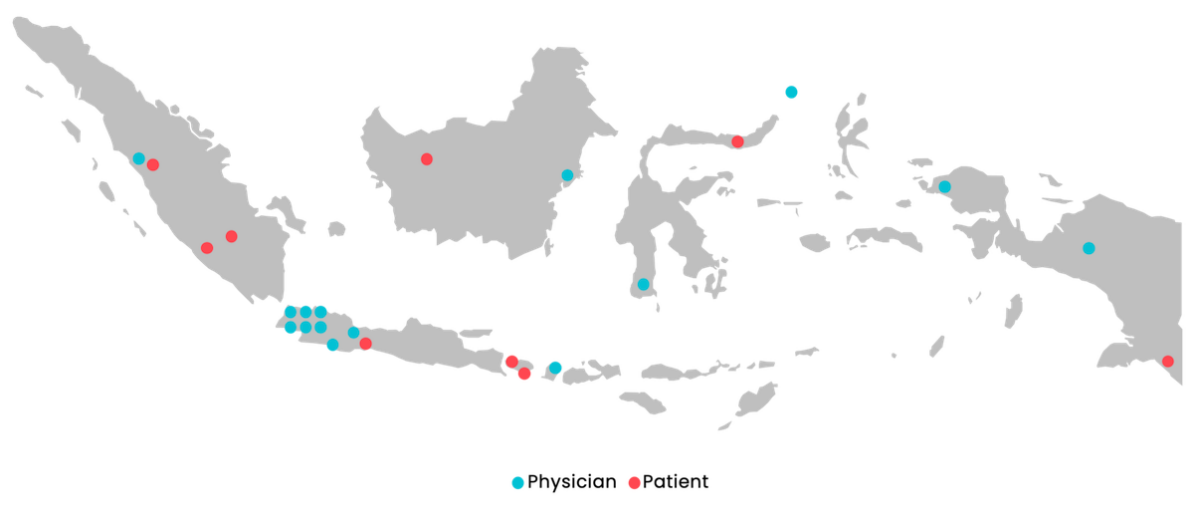
Distribution of physician and patient interviews across Indonesia.

Figure 2 illustrates the geographic distribution of study participants across Indonesia. Physicians and patients are represented according to their respective regions. This highlights the coverage across 6 major regions and shows regional representation.

### Physicians’ Perspectives

#### Overview

The study involved 15 physicians from 6 regions in Indonesia, as illustrated in Figure 2, representing a range of specialties: general practice (n=5), immunology (n=4), rheumatology (n=3), plastic surgery (n=1), pediatrics (n=1), and ophthalmology (n=1). Participants’ clinical experience varied, with 2 participants having over 20 years of practice, 6 having 16‐20 years, 2 having 6‐10 years, and 5 having between 1‐5 years of experience.

Additionally, the physicians practiced across all hospital classifications, as defined in Article 340/MENKES/PER/III/2010, which categorizes hospitals based on service capabilities, infrastructure, and available resources [74-76]. The classification system categorizes hospitals into four types: class A, B, C, and D. Class A hospitals, primarily located in major cities, serve as top referral centers with extensive facilities and a wide range of medical and subspecialty services. Class B hospitals, mostly found in regional areas, provide at least 11 specialist and limited subspecialist services. Class C hospitals focus on essential specialist care and are equipped with a minimum number of medical specialties. Class D hospitals, commonly referred to as Community Health Centers (Pusat Kesehatan Masyarakat), offer basic medical services with limited specialized care.

#### Evolution of Telemedicine Adoption

Before the COVID-19 pandemic, telemedicine adoption in Indonesia was sporadic and limited. Among 15 physicians surveyed, 12 reported using telemedicine. Additionally, 1 physician recalled that “telemedicine didn’t exist yet” around 2015, reflecting the nascent state of digital health at the time.

The pandemic became a turning point. A total of 11 out of 15 physicians began using virtual platforms such as WhatsApp (Meta Platforms, Inc), Zoom (Zoom Communications, Inc), standalone health tech apps like HaloDoc (PT Media Dokter Investama) and Alodokter (PT Alodokter Teknologi Indonesia), as well as hospital-based telemedicine systems [65-68]. During this period, telemedicine was particularly valuable for triaging patients, conducting follow-ups, and managing medications. The need to reduce physical contact due to infection risks made remote consultations not just practical but essential. Many hospitals and clinics swiftly adopted telemedicine services, though often with basic tools or improvised infrastructure.

As COVID-19 cases declined, physicians observed a reduction in telemedicine usage as patients gradually returned to in-person visits. Still, telemedicine continues to serve an important role, especially for follow-up care and for managing chronic or rare conditions. This transition is reflected in 1 physician’s experience:

At one point, I was handling up to 23 patients a day, but during a single Zoom call, there could be five family members present. Afterward, I would continue treating the rest of my patients. That was mostly in 2019‐2020, but then the numbers started declining. Post-COVID, it’s been fewer, I primarily manage autoimmune patients from remote areas.

[D13]

While full replacement of in-person care remains unlikely, the pandemic has established telemedicine as a lasting complement within hybrid care models. Half of the physicians viewed it as unsuitable for initial diagnoses, but they now use it for monitoring, education, second opinions, and psychological support. This approach is particularly effective for extending care and reducing financial burdens to patients in remote locations. In this context, pediatric cardiologists in Jakarta conduct monthly follow-ups via video consultation with patients in Aceh, located in the westernmost part of Indonesia. Given the long waiting times and the need for continuous monitoring, a process known as the surgical conference is used to assess and prioritize patient conditions. One physician shared:

They are usually asked to have monthly follow-ups via the online clinic. They also check the status of the surgical conference queue. So, it just keeps moving along.

[D4]

#### Challenges

In total, 14 out of 15 physicians identified the lack of physical examination as a key limitation in telemedicine, as noted:

However, if I’ve never met a patient in person, never done a physical examination, or never had direct contact, it’s challenging to diagnose complex diseases [...] for diagnoses that require physical examinations, telemedicine can’t fully replace in-person visits.

[D7]

Telemedicine has also increased workloads. Two physicians reported burnout due to additional consultation demands. A physician explained:

Right now, telemedicine is just a replacement for face-to-face consultations. It does not reduce the workload; it adds to it. [...] So, telemedicine adds extra time slots. To reduce burnout, we need more doctors and better networking.

[D7]

Legal uncertainty adds another layer of concern. Five physicians questioned data ownership when using third-party apps, emphasizing “[a]nother issue is significant patient privacy concerns regarding data security and encryption measures” (D9).

#### Future Directions

Telemedicine is advancing toward greater accessibility, efficiency, and diagnostic accuracy. A key innovation is the creation of specialized hubs linking medical teams across hospitals. As explained by a physician, this hub-and-spoke model is underway at his or her hospital, beginning with neurosurgery:

For example, at our hospital, what we plan for the future is to have this. Currently, our neurosurgery team is already working on it. The main hub for the neurosurgery team is at our hospital. But then, in every hospital branch, there is a hub that becomes part of the neurosurgery network.

[D7]

Another promising development is artificial intelligence (AI)–assisted diagnostics. A physician highlighted a project targeting diabetic retinopathy:

We are currently developing AI tools for diabetic retinopathy [...] The AI analyzes the photos to categorize them automatically [...] In remote regions, access to equipment is still a challenge.

[D8]

Additionally, a physician cited virtual reality (VR) integration in Japan to support remote assessments:

As currently being implemented at the University of Nagasaki, Japan [...] Patients visit a clinic on a remote island, and physicians remotely assess their condition using VR.

[D15]

### Patients’ Perspectives

#### Participant Characteristics

The data is based on interviews with 9 patients diagnosed with various autoimmune conditions, including rheumatoid arthritis, systemic lupus erythematosus, Sjögren’s disease, psoriasis, antiphospholipid syndrome, Graves’ disease, and scleroderma, with time since diagnosis ranging from 2 to 17 years. Participants, aged between 26 and 59 years, came from diverse professional backgrounds and lived in both urban and rural areas. Seven were covered by Indonesia’s national health insurance (BPJS), while 2 financed their health care independently.

#### Telemedicine Adoption Shaped by Health Care Access

Patients with autoimmune diseases in Indonesia face significant disparities in health care access. Accurate diagnosis typically requires referral to class A hospitals located in major cities, due to the scarcity of subspecialists in rural or outer island regions. The average diagnosis delay exceeded 1 year. One patient was diagnosed in Malaysia due to the lack of domestic expertise during the early 2000s, as local specialists were unavailable at the time.

While some patients now receive initial evaluations at class B hospitals, advanced care still necessitates travel to central facilities. A patient from eastern Indonesia reported a 400 km journey, taking 10 hours by bus through mountainous terrain, costing up to IDR 10 million (€580) per visit.

The eight to ten hours by land is really far, especially with the poor infrastructure with mountain roads and winding paths [...], so my mother called instead. There is no consultation fee; only the medicine has a cost, but it is expensive.

[P6]

Due to the physical and financial burden, the patient was later transferred to a nearer class B hospital, albeit with limited specialist support.

Telemedicine has emerged as a critical solution for bridging these access gaps. Among the 9 patients interviewed, those living in urban or semiurban areas within 5 km of a hospital preferred in-person care, citing convenience and minimal travel time.

The distance is probably one kilometer. You can walk or ride a motorbike. It usually takes about 5-10 minutes by motorbike.

[P9]

In contrast, 6 patients residing over 50 km from the nearest hospital relied heavily on telemedicine for follow-ups and flare-ups. Poor infrastructure meant that a 50 km trip could take up to 3 hours, pushing patients to communicate directly with their physicians via WhatsApp text and video calls. This is an informal, yet reliable channel in the absence of more robust telehealth systems.

I once had a check-up, and everything was fine, with no complaints for a month. Then, just a week after the check-up, I suddenly experienced pain in my hand, swelling, and an unusual toothache. When I consulted a dentist, they found no inflammation, but the pain persisted. [...] I messaged the doctor directly and sent a photo of the dental examination results.

[P2]

Notably, 3 patients with multiple chronic conditions reported using WhatsApp video calls during emergencies to replace in-person visits. They maintained direct contact with their physicians with whom they had been in care for more than 5 years.

I was able to video call my personal doctor directly via WhatsApp while I was in isolation due to COVID-19 to discuss my symptoms along with my autoimmune conditions. The consultation lasted 10‐15 minutes and was free of charge.

[P5]

Telemedicine was also used for prescription renewals and noncritical follow-ups. Two patients receiving care at specialized autoimmune clinics reported being notified via WhatsApp when their condition was stable, skipping the usual in-person appointment. Interestingly, none of the patients used commercial health-tech apps (eg, Halodoc), preferring direct contact with trusted physicians. As 1 patient explained:

I already know the doctor, so I just message them directly.

[P2]

In remote regions where specialist access remains sparse and health literacy is rising, patients have begun turning to social media for medical advice. This noted the role of social media in improving access to medical information and direct physician interaction [66,67].

I asked him on Instagram or TikTok, “Doctor, is there an alternative to Myfortic because it’s hard to find?”

[P1]

Together, these findings highlight how geography, infrastructure, and personal networks shape health care access and telemedicine use. Patients in remote areas, particularly those with long-term physician relationships, increasingly rely on digital tools to overcome systemic barriers to consistent and affordable care.

#### Cost-Effectiveness

Telemedicine significantly reduced financial burdens for patients requiring frequent monitoring. Of the 9 participants, 6 reported measurable cost savings after switching from monthly in-person visits to a hybrid model with 1 in-person visit and 5 virtual consultations over 6 months.

At the time of the study, the exchange rate was approximately €1=17,400 IDR (US $1=IDR 15,500 [2024 average]) [77,78]. The average cost per in-person visit was IDR 2 million (€115), with transportation and accommodation expenses increasing the total cost substantially for remote patients. In comparison, teleconsultations conducted via WhatsApp were free. Table 2 below summarizes the financial impact, as reported by multiple participants in this study.

These results reflect a cost reduction of 65%, from IDR 42.0 million (€2484) to IDR 16.5 million (€869), corresponding to an absolute savings of IDR 25.5 million (€1615), without compromising care quality for stable patients. However, public insurance (BPJS) does not fully cover all lab tests or medications. Out-of-pocket costs varied from IDR 200,000 (€12) to IDR 7 million (€405) per month, depending on the disease progression. One patient noted paying IDR 7 million monthly during the first year post diagnosis due to uncovered medications.

Hence, telemedicine offers significant financial and logistical relief for patients in eastern Indonesia, reducing the need for costly and exhausting multileg hospital trips. Thus, telemedicine is highly valued, not only for its economic advantages but also for its emotional and physical relief.

**Table 2.** Exemplary comparison of cost savings based on patient interviews.

Category	In-person visits	Hybrid model	Cost savings
Visit frequency (6 mo)	6 in-person visits (monthly)	1 in-person visit+5 telemedicine consultations	—^a^
Cost per in-person visit (IDR)^b^	2,000,000	2,000,000	0
Total in-person visit cost (IDR)	12,000,000 (IDR 2,000,000x6)	2,000,000 (1 visit)	10,000,000
Telemedicine consultation cost (IDR)	0	0 (via WhatsApp)^c^	0
Medication costs (IDR)	9,000,000 (IDR 1,500,000 x 6)	9,000,000 (IDR 1,500,000 x 6)	0
Transportation costs (IDR)	12,000,000 (round-trip for all visits)	2,000,000 (1 trip)	10,000,000
Additional costs (accommodation, meals) (IDR)	9,000,000 (overnight stays, meals)	1,500,000	7,500,000
Total cost (IDR)	42,000,000	16,500,000	25,500,000 saved

a Not applicable.

b All costs are reported in Indonesian Rupiah (IDR). Conversion to US dollars (US $) was performed using an exchange rate of US $1=IDR 15,500 (2024 average).

c Free, under specific conditions (eg, a close physician-patient relationship, typically established for at least 5 years since the initial diagnosis).

## Discussion

### Overview

This study examines the impact of telemedicine adoption on the management of chronic and rare diseases in Indonesia. Telemedicine has demonstrated significant potential in enhancing chronic illness care and has been recognized as a critical component of Indonesia’s health care response during the COVID-19 pandemic [79-81]. By exploring the perspectives of both patients and health care professionals, this research offers valuable insights into the effectiveness, challenges, and opportunities of telemedicine for managing chronic and rare diseases in the postpandemic era.

### Principal Findings and Alignment With Prior Literature

The results confirm existing theoretical insights into telemedicine adoption, particularly in Indonesia. The findings align with previous studies, which highlight both synchronous (real-time consultations) and asynchronous (store-and-forward) telemedicine models [16,18]. Physicians’ reliance on WhatsApp, Zoom, and hospital-based platforms demonstrates the transition from minimal telemedicine adoption prepandemic to a more structured implementation during and post–COVID-19.

However, the results reveal that despite growing adoption, telemedicine is still not evenly distributed. This reflects reports that, although telemedicine adoption increased during the pandemic, its growth has since plateaued or declined in some regions [22,23]. The Indonesian data aligns particularly well with the trend observed in India, where slow adoption is due to remote infrastructure challenges.

**Figure 3.**
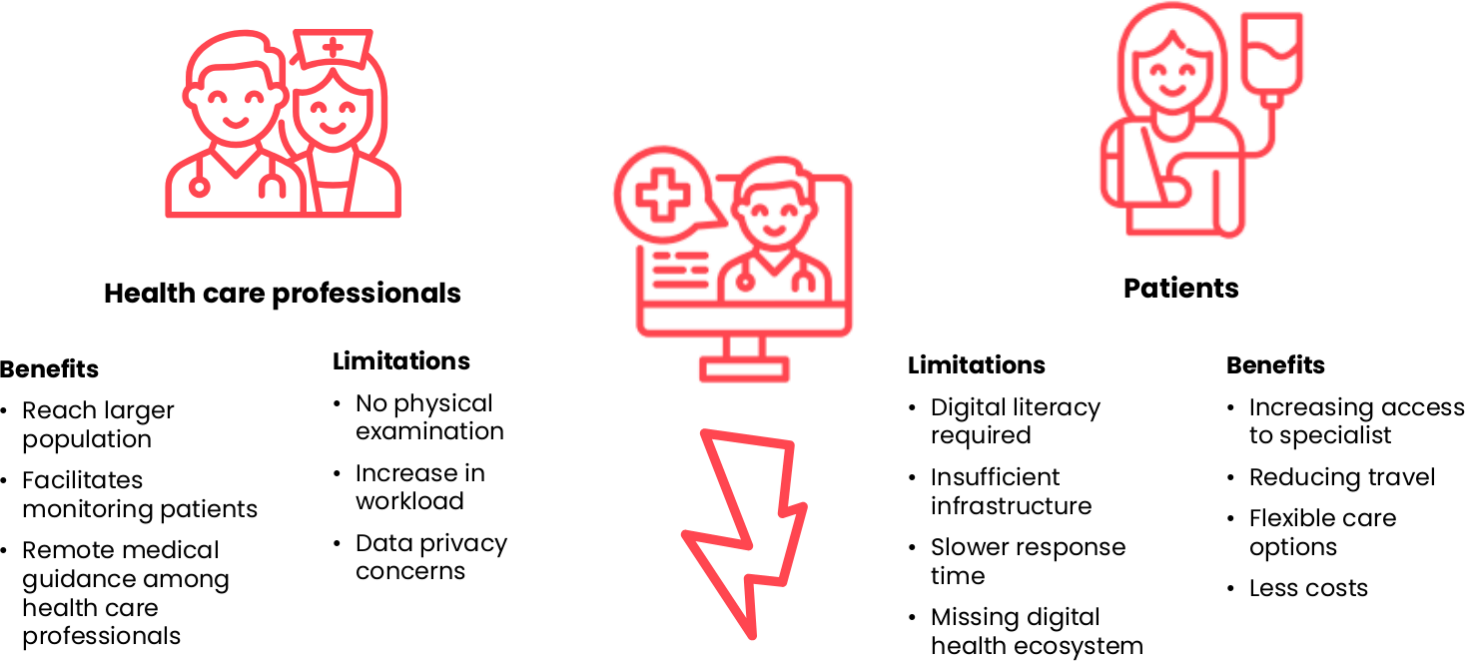
Summary of the benefits and limitations of telemedicine in Indonesia.

From the perspectives of both patients and health care providers, telemedicine was associated with improved access to care, reduced travel time and costs, and was particularly useful for follow-ups and managing chronic diseases. However, shared concerns included clinical limitations, unreliable internet connections, and legal uncertainties. Referring to Figure 3, health care professionals benefit from expanded patient reach and improved remote monitoring but face limitations such as the inability to conduct physical examinations and increased administrative burdens. On the patient side, benefits like improved access to specialists and reduced travel are offset by challenges such as low digital literacy and poor internet access.

However, this analysis may not fully capture variations across rural Indonesia. Some areas, particularly those with digitally literate younger chronic patients and stable internet access, face physician shortages rather than technological barriers. In such cases, patients often turn to social media messaging platforms to seek timely medical guidance from appropriate specialists, reflecting the evolving role of asynchronous telemedicine in bridging health care gaps [49,51,66]. Telemedicine is most effective and feasible for patients with high digital literacy, stable internet access, and low disease activity [43]. This further supports the trend in Indonesia, where telemedicine serves as a viable alternative for patients who meet these criteria but lack immediate access to physicians. Our study contributes that access to medical specialists is very limited outside the capital, and that telemedicine could significantly improve care across the country by improving access and lowering cost. Regarding chronic and rare disease management, the physicians’ insights reflect the Asia-Pacific League of Associations for Rheumatology recommendations that telemedicine is effective for follow-ups but should not replace face-to-face visits for initial diagnoses [52]. This aligns with findings that emphasize the importance of physical examinations in clinical decision-making [49]. Moreover, the results echo research showing that over half of autoimmune patients prefer in-person consultations, particularly for first-time visits [43]. However, once a patient-physician relationship is established, they are more open to telemedicine follow-ups, reflecting evidence that patients prefer continuity with familiar physicians [15].

The cost-effectiveness of telemedicine in chronic disease management is also validated. Telemedicine has been shown to reduce travel costs and improve medication adherence, a pattern echoed in physicians’ reports of its benefits for remote patients in Kalimantan and Sulawesi [45,47]. These results reinforce previous findings that telemedicine reduces costs for patients and providers, reducing inequalities in health care delivery and thus advancing SDGs 3 and 10. A systematic review of studies conducted in high-income countries further indicates that patients with chronic diseases show a measurable willingness to pay for telemedicine, ranging between 19% and 70% across studies, with higher willingness linked to greater distance from health facilities, while older patients were less willing to pay [82]. In Sweden, digital consultations led to a 41.5% reduction in health care expenses [54], a trend mirrored in Indonesia, where chat-based teleconsultations are considerably more affordable than face-to-face visits [83,84]. However, the study also highlights barriers to affordability. Unlike Sweden and France, where telemedicine consultations are reimbursed by statutory health insurances, Indonesia’s system still relies on private insurance or out-of-pocket payments [55,85]. This limits access for low-income populations and supports concerns that telemedicine may exacerbate health care inequalities if not implemented equitably [49,86].

Beyond cost savings, telemedicine improves access to specialists for patients in remote areas who require consultations with rare disease specialists without geographical constraints [87,88]. This is evident in Indonesia’s surgical conference model, where pediatric cardiologists conduct video consultations to assess and monitor children awaiting congenital heart disease surgery. Therefore, this study confirms existing challenges from the literature.

### Practical Implications: Potential for Future Telemedicine Advancement

While telemedicine presents substantial benefits, physicians continue to recognize its limitations, particularly the necessity of physical examinations for certain medical fields such as rheumatology, neurology, and surgery [89]. Although effective for initial screenings and follow-ups, these specialties often require in-person assessments. Additionally, concerns have been raised about physician burnout, as virtual consultations expand patient reach beyond local boundaries and increase workload [59,90]. While the phenomenon of an increased workload and the resulting risk of burnout is not new to health care professionals, it certainly needs more attention in the context of telemedicine, from scholars and regulators alike. Similarly, concerns have been raised in the Association of Southeast Asian Nation-Japan Center report, where Indonesian health care providers express uncertainty over data security and ownership when using independent telemedicine platforms [91]. While unregulated platforms such as WhatsApp and Zoom are widely used at the moment (also in other health-related contexts such as nutrition monitoring), it remains to be seen whether regulators will enforce other platforms in the future to increase privacy and security, while maintaining low entry barriers and an easy-to-use system [92]. Furthermore, infrastructural challenges—specifically unreliable electricity and limited internet connectivity in remote areas—continue to hinder telemedicine implementation in rural Indonesia [6,33].

Despite these challenges, the findings point to significant opportunities for advancing telemedicine. Physicians’ vision for the development of telemedicine hubs across Indonesia aligns with Sweden’s integrated telehealth model, which connects regional hospitals and primary care centers with specialized institutions to optimize care delivery [26]. Technological innovations such as VR can enhance remote diagnostics and treatment by offering immersive patient experiences, particularly beneficial in underserved regions [16,93]. Moreover, AI-assisted tools, including automated retinal screening for diabetic retinopathy, show promise in increasing diagnostic accuracy and early intervention. Integrating such tools into national health programs like Program Pengelolaan Penyakit Kronis could significantly strengthen chronic disease management, especially for diabetes, by facilitating proactive care and reducing complications [30].

### Study Limitations and Directions for Future Research

While this study provides valuable insight into telemedicine implementation in a low-resource setting such as Indonesia, it is not without limitations. First, the sample size and scope were limited to a specific group of physicians and patients, which may not fully capture the diversity of Indonesia’s health care landscape. Although our qualitative analysis did not reveal notable differences in telemedicine use based on gender or socioeconomic status within the small patient sample, we acknowledge that the composition of the sample was limited and skewed. Most of the participants were female and were predominantly insured by BPJS. Second, the study did not sufficiently account for regional disparities. Although adoption trends were discussed, variations in infrastructure quality, digital literacy, and health care accessibility between urban and rural areas were not comprehensively addressed. These disparities significantly influence the rate of telemedicine uptake and its overall effectiveness. Third, the study adopts a primarily short-term perspective, focusing on current patterns of telemedicine use without evaluating its long-term impact on patient outcomes, health care systems, or cost-efficiency. Longitudinal studies are needed to assess whether telemedicine can sustainably support health care delivery in Indonesia over time. At last, while the study acknowledges certain challenges related to technology and policy, it does not provide an in-depth analysis of regulatory environments, reimbursement structures, or technological readiness. These factors are crucial for understanding the scalability and sustainability of telemedicine services.

Future research should aim to expand sample diversity by including a wider range of health care professionals, patient populations, facility types, and geographic regions. In particular, studies with larger and more demographically diverse participant groups are needed to explore potential variations in telemedicine access, use, and user experience across gender, income, and insurance categories. This broader scope would enable a more representative and comprehensive understanding of telemedicine adoption across Indonesia. Additionally, long-term studies are necessary to evaluate the effects of telemedicine on patient health outcomes, cost-effectiveness, and physician workload. Exploring alternatives to in-person examinations, such as VR-based assessments, as trialed in Japan, may offer innovative solutions to address diagnostic challenges in remote settings. Furthermore, a detailed analysis of policy frameworks, legal regulations, data security protocols, and funding mechanisms is essential to assess their influence on telemedicine integration. Comparative studies with countries facing similar health care challenges could also provide valuable benchmarks and help identify best practices for more effective and equitable telemedicine implementation in Indonesia.

### Conclusions

The findings of this study align with existing literature while providing additional insights into the regional disparities and infrastructure challenges related to telemedicine adoption in a major South-East Asian country. A noticeable shift in telemedicine usage occurred before, during, and after the COVID-19 pandemic. Prior to the pandemic, telemedicine adoption was minimal, with limited engagement from both physicians and patients. During the pandemic, there was a significant increase in the use of telemedicine platforms, as remote consultations became essential for managing patient volumes through tools like WhatsApp, Zoom, independent health technology applications, and hospital-based systems. Therefore, targeted investment in digital infrastructure and internet connectivity, particularly in underserved regions, would help reduce geographic inequities.

Postpandemic, the use of telemedicine for general consultations has decreased, though it continues to be important for follow-up care, the management of chronic and rare diseases, and improving access to specialists in remote areas, thereby supporting progress toward SDGs 3 and 10. While telemedicine has contributed to increased health care accessibility and cost-effectiveness, challenges remain, such as inadequate infrastructure, physician burnout, legal uncertainties, and the inability to conduct physical examinations remotely, which can hinder its impact on health equity. Hence, implementing support mechanisms for health care professionals, such as workload management systems and mental health progress, is crucial for long-term adoption.

Emerging models, such as telemedicine hubs, VR-based diagnostics, and AI-assisted screening, offer potential solutions to bridge these gaps and further promote equitable health care delivery in line with SDG 3 and SDG 10. However, the long-term success and broader adoption of telemedicine in Indonesia require sustainable regulatory frameworks, adequate funding models, consideration of the well-being of health care professionals, and continued investment in digital infrastructure. Addressing these challenges will be essential to maximize telemedicine’s contribution to more inclusive and resilient health care systems that advance global commitments to SDG 3 and 10.

## Supplementary material

10.2196/83462 Multimedia Appendix 1 Qualitative research design.

10.2196/83462 Checklist 1 COREQ checklist.

## Abbreviations

AI: artificial intelligence
BPJS: Badan Pengelola Jaminan Sosial Kesehatan (National Health Insurance Agency)
COREQ: Consolidated Criteria for Reporting Qualitative Research
GAI: generative artificial intelligence
NCD: noncommunicable disease
SDG: sustainable development goal
VR: virtual reality
