# Supplementary material for: Telemedicine Adoption for Managing Chronic and Rare Diseases in Indonesia During and Beyond the COVID-19 Era: Qualitative Study
Source: J Med Internet Res. 2026 Mar 19;28:e83462. doi: 10.2196/83462 (PMC13002011; doi:10.2196/83462)
Supplement: Multimedia Appendix 1 [file jmir-v28-e83462-s001.docx]

# Appendix

#### Appendix S1. Interview Guide for Physicians

| **Section** | **Topic** | **Questions** | **Frequency of Respondents** |
| --- | --- | --- | --- |
| **Introduction** | Researcher introduction | 1. Brief introduction and the purpose of the research. 2. Explain the study aims to explore the role of telemedicine in managing chronic and rare disease from perspective of healthcare professionals. 3. Assure confidentiality and obtain verbal consent to record the session. |  |
| **Section 1:**  Current medical practice | General background | 1. Could you share your professional background and current role in healthcare? 2. How many years have you been practicing medicine? 3. What is your specialty and main area of expertise? 4. Where is your current practice located? |  |
|  | Professional background | 1. What led you to specialize in your current medical field? 2. What illnesses or conditions do you handle most often? |  |
|  | Experience with digital health | 1. Have you used digital health technologies before telemedicine? |  |
| **Section 2:**  Telemedicine usage | General use of telemedicine | 1. Have you had prior experience using telemedicine in your practice? 2. In your opinion, how has telemedicine changed the way healthcare is delivered in your field? 3. Could you describe how often and in what ways you use telemedicine for patient care? 4. What platform or system do you use for telemedicine consultations (e.g., WhatsApp, hospital apps, independent telehealth platforms)? |  |
|  | Types of cases managed via telemedicine | 1. What types of diseases or conditions do you find suitable for telemedicine consultations? 2. Are there specific cases where telemedicine is more effective than in-person visits? 3. How do you determine whether a patient should be seen via telemedicine or in person? |  |
| **Section 3:**  Benefits and challenges of telemedicine | Perceived benefits | 1. What are the main advantages of telemedicine in managing chronic and rare diseases? 2. Has telemedicine helped improve patient adherence to treatment plans? If so, have you observed any improvements in patient monitoring and follow-up through telemedicine? |  |
|  | Challenges and limitations | 1. What are key challenges you have faced while using telemedicine? 2. Have you encountered any difficulties related to internet connectivity, platform usability, or patient literacy? 3. How do you handle cases where a physical examination is necessary, but the patient is consulting remotely? |  |
| **Section 4:**  Telemedicine and healthcare access | Geographical and socioeconomic considerations | 1. Do you think telemedicine is effectively addressing healthcare disparities in remote or underserved areas? 2. How do financial factors (e.g., BPJS, private, insurance, self-pay) impact patients’ access to telemedicine services? |  |
|  | Telemedicine in the post-pandemic era | 1. Has the adoption of telemedicine changed after the COVID-19 pandemic? If so, how? 2. Do you think telemedicine will continue to grow, or do you see a decline in its use? |  |
| **Section 5:**  Future of telemedicine | Potential developments | 1. What do you think the future of telemedicine looks like in your field? 2. Are there any emerging technologies (e.g., AI, remote monitoring devices) that you believe could enhance telemedicine effectiveness? |  |
| **Closing questions** | Final thoughts and recommendations | 1. Is there anything else you would like to add regarding telemedicine and its impact on patient care? 2. Do you have any recommendations for improving telemedicine for both healthcare professionals and patients? 3. Would you recommend another physician to take part in this study? |  |

#### Appendix S2. Physician Overview

| **Physician** | **Specialty** | **Geographical Unit** | **Years of Experience** | **Use Telemedicine** |
| --- | --- | --- | --- | --- |
| D1 | Rheumatologist | Kalimantan | 33 | Yes |
| D2 | Immunologist | Java | 18 | Yes |
| D3 | General Practitioner | Java | 4 | Yes |
| D4 | General Practitioner | Nusa Tenggara Islands | 2 | Yes |
| D5 | Plastic, Reconstructive, and Aesthetic Surgeon | Java | 10 | Yes |
| D6 | General Practitioner | Papua | 6 | No |
| D7 | Immunologist | Java | 17 | Yes |
| D8 | Ophthalmologist | Java | 17 | Yes |
| D9 | Pediatrician | Papua | 10 | No |
| D10 | General Practitioner | Java | 2 | Yes |
| D11 | Rheumatologist | Java | 25 | Yes |
| D12 | Immunologist | Sumatra | 16 | No |
| D13 | Immunologist | Sulawesi | 22 | Yes |
| D14 | General Practitioner | Sulawesi | 2 | Yes |
| D15 | Rheumatologist | Java | 16 | Yes |

#### Appendix S3. Interview Guide for Patients

| **Section** | **Topic** | **Questions** |
| --- | --- | --- |
| **Introduction** | Researcher introduction | 1. Brief introduction and the purpose of the research. 2. Explain the study aims to explore the role of telemedicine in managing chronic and rare disease from perspective of healthcare professionals. 3. Assure confidentiality and obtain verbal consent to record the session. |
| **Section 1:**  Demographic factors | General background | 1. Could you share your age, gender, occupation, and where you live? |
| **Section 2:**  Medical background | Illness type and diagnosis | 1. What conditions have you been diagnosed with (chronic, autoimmune, rare)? 2. How long were you diagnosed? |
|  | Treatment journey | 1. Have did your treatment journey start? 2. Has your treatment approach changed over time? 3. How has your medical condition affected your daily life? 4. What specialists handle your case? |
| **Section 3:**  Telemedicine adoption | Usage status | 1. Have you ever used telemedicine for managing your condition? If yes, when did you start? if no, what are the reasons? |
|  | Platform used | 1. What telemedicine platforms do you use (e.g., WhatsApp, hospital apps, independent healthtech apps)? 2. How often do you use telemedicine (e.g., for routine check-ups, emergencies)? |
| **Section 4:**  Healthcare access and costs | Previous treatment | 1. Where did you previously receive treatment (hospital name, location, and class)? 2. What was your payment type (BPJS, private insurance, self-pay)? 3. What transportation did you use to reach the hospital? 4. How much did your medical and additional costs (e.g., accommodation, meals) amount to? |
|  | Ongoing treatment | 1. How has your treatment changed since using telemedicine? 2. Are you still visiting hospitals or relying more in telemedicine? 3. What are the financial differences between your previous and current treatment approach? |
| **Section 5:**  Challenges and barriers | Travel and infrastructure issues | 1. Have you experienced difficulties reaching healthcare facilities due to travel burden or infrastructure limitations? |
|  | Availability of physicians | 1. Have you faced challenges in accessing specialists for your conditions? 2. Do you think telemedicine helps bridge this gap? |
|  | Cost and insurance barriers | 1. Have you encountered any financial limitations in accessing telemedicine or traditional healthcare? 2. How does your insurance coverage affect your healthcare access? |
|  | Digital literacy and accessibility | 1. Do you find telemedicine platforms easy to use? 2. Have you experienced any technical difficulties? |
| **Section 6:**  Patient perceptions and experience | Satisfaction levels | 1. How satisfied are you with telemedicine consultations? 2. Have you had positive or negative experiences with remote healthcare? |
|  | Healthcare continuity | 1. Do you feel that telemedicine helps maintain continuity in your treatment? 2. How has it impacted your ability to receive care when needed? |
|  | Preference for telemedicine vs. in-person care | 1. Do you prefer telemedicine or in-person visits for managing your conditions? Why? 2. In what situations do you feel telemedicine is most helpful? |
| **Closing questions** | Final thoughts and reflections | 1. Is there anything else you would like to share about your experience with telemedicine? |

#### Appendix S4. Patient Overview

Demographic Data

| **Patient** | **Gender** | **Age** | **Geographical Unit** | **Use Telemedicine** | **Payment Type** |
| --- | --- | --- | --- | --- | --- |
| P1 | Female | 29 | Kalimantan | Yes | BPJS |
| P2 | Female | 47 | Java | Yes | BPJS |
| P3 | Female | 59 | Nusa Tenggara Islands | Yes | BPJS |
| P4 | Female | 57 | Sumatra | Yes | Self-pay |
| P5 | Female | 38 | Sumatra | Yes | BPJS |
| P6 | Male | 30 | Sulawesi | No | BPJS |
| P7 | Female | 26 | Sumatra | Yes | BPJS |
| P8 | Female | 46 | Nusa Tenggara Islands | Yes | Self-pay |
| P9 | Female | 28 | Papua | No | BPJS |

Medical Treatment History

| **Patient** | **Previous Treatment** | | | | | | | **Ongoing Treatment** | | | | | | | | **Remarks** | |
| --- | --- | --- | --- | --- | --- | --- | --- | --- | --- | --- | --- | --- | --- | --- | --- | --- | --- |
|  | **Hospital Class*** | **Physician** | **Distance to Hospital (km)** | **Travel Time One-Way (Hour)** | **Control Period (Month)** | **Total Costs** | | **Hospital Class*** | **Physician** | **Distance to Hospital (km)** | **Travel Time One-Way (Hour)** | **Control Period (Month)** | **Total Costs** | |  | |  |
|  |  |  |  |  |  | **IDR** | **EUR** |  |  |  |  |  | **IDR** | **EUR** |  |  |  |
| P1 | A | Rheumatologist | 1.000 | 16 | 3 | 5.100.000 | 305 | B | Internist | N/A | 0,25 | 1 | 50.000 | 3 | Transferred to the nearest class B hospital | |  |
| P2 | A | Rheumatologist | 75 | 3 | 1 | 600.000 | 36 | Consistent with previous treatment | | | | | | | No changes | |  |
| P3 | A | Rheumatologist | 3 | 0,25 | 1 | 250.000 | 15 | Consistent with previous treatment | | | | | | | No changes | |  |
| P4 | C (Private clinic) | Rheumatologist, Oncologist | 790 | 2 | 1 | 5.000.000 | 299 | Consistent with previous treatment | | | | | | | Hybrid model | |  |
| P5 | A | Immunologist, Rheumatologist | 321 | 8 | 3 | 4.000.000 | 239 | C | Internist | 50 | 1,5 | 1 | 1.300.000 | 78 | Transferred to the nearest class B hospital | |  |
| P6 | A | Immunologist, Rheumatologist | 790 | 10 | 1 | 10.000.000 | 597 | B | Internist | N/A | 1 | 1 | 3.050.000 | 182 | Transferred to the nearest class B hospital | |  |
| P7 | A | Rheumatologist | 91 | 2 | 1 | 450.000 | 27 | B | Internist | N/A | 0,5 | 1 | 50.000 | 3 | Transferred to the nearest class B hospital | |  |
| P8 | B | Rheumatologist | 73 | 3 | 1 | 1.500.000 | 90 | C (Private clinic) | Rheumatologist | 73 | 3 | 1 | 1.500.000 | 90 | Switched to the nearest private clinic | |  |
| P9 | B | Internist | 5 | 0,5 | 1 | 40.000 | 2 | Consistent with previous treatment | | | | | | | No changes | |  |

*Class A: Major urban hospitals with comprehensive facilities and full subspecialty services; Class B: Regional hospitals with at least 11 specialist and limited subspecialty services; Class C: Hospitals offering essential specialist care with basic medical specialties; Class D: Community Health Centers (Puskesmas) providing basic medical services with minimal specialization.

#### Appendix S5. Physicians’ Perspective Coding Framework

| Physicians' perspectives on telemedicine  │  ├── General experience  │ ├── Years in practice  │ ├── Specialization and expertise  │ ├── Exposure to digital health technologies  │ ├── Previous experience with remote consultations  │ └── Attitudes toward telemedicine  │  ├── Telemedicine usage  │ ├── Frequency of use  │ ├── Platforms used (WhatsApp, hospital apps, independent telehealth apps)  │ ├── Type of cases managed  │ └── Decision-making for telemedicine vs. in-person care  │  ├── Benefits of telemedicine  │ ├── Monitoring stable patients  │ ├── Accessibility for remote patients  │ ├── Screening and initial advice  │ ├── Cost-effectiveness (time and money)  │ ├── Medication and prescription management  │ └── Reduced hospital overcrowding  │  ├── Challenges in telemedicine  │ ├── Diagnosis and treatment difficulties  │ ├── Technical issues (internet connectivity, platform usability)  │ ├── Physician readiness and burnout  │ ├── Legal, privacy, and data security concerns  │ └── Patient compliance and digital literacy  │  ├── Healthcare accessibility  │ ├── Geographical disparities  │ ├── Impact of financial barriers (BPJS, private insurance, self-pay)  │ └── Referral processes and system limitations  │  └── Telemedicine trends and adoption  ├── Pre-, during-, and post-pandemic adoption trends  ├── Future outlook of telemedicine  ├── Emerging technologies (AI, remote monitoring devices)  ├── Condition-specific effectiveness  │ ├── NCDs  │ ├── Psychiatric Care  │ ├── Autoimmune Rheumatic Diseases  │ ├── Dermatology  │ ├── Influenza/Common Cold  │ └── Others  └── Policy and institutional recommendations |
| --- |

####

#### Appendix S6. Patients’ Perspective Coding Framework

| Patients’ perspectives on telemedicine  │  ├── Demographic factors  │ ├── Age  │ ├── Gender  │ ├── Occupation  │ └── Region  │  ├── Medical background  │ ├── Illness type (chronic, autoimmune, rare)  │ ├── Years since diagnosis  │ ├── Treatment journey  │ └── Specialists handling patient’s case  │  ├── Telemedicine adoption  │ ├── Usage status (uses vs. does not use)  │ └── Platforms used (WhatsApp, hospital apps, healthtech independent apps)  │  ├── Healthcare access and costs  │ ├── Previous treatment  │ │ ├── Hospital name, location, and class  │ │ ├── Payment type (BPJS, private insurance, self-pay)  │ │ ├── Transportation (type, distance, cost)  │ │ └── Medical and additional costs  │ │  │ └── Ongoing treatment  │ ├── Hospital name, location, and class  │ ├── Payment type (BPJS, private insurance, self-pay)  │ ├── Transportation (type, distance, cost)  │ └── Medical and additional costs  │  ├── Challenges & barriers  │ ├── Travel burden and infrastructure issues  │ ├── Limited availability of specialists  │ ├── Cost constraints and insurance limitations  │ └── Digital literacy and telemedicine accessibility  │  └── Patient perceptions & experience  ├── Observation on telemedicine experience (positive, neutral, negative)  ├── Impact on healthcare continuity  └── Preference for in-person vs. telemedicine |
| --- |
