# Supplementary material for: Telemedicine Adoption for Managing Chronic and Rare Diseases in Indonesia During and Beyond the COVID-19 Era: Qualitative Study
Source: J Med Internet Res. 2026 Mar 19;28:e83462. doi: 10.2196/83462 (PMC13002011; doi:10.2196/83462)
Supplement: Checklist 1 [file jmir-v28-e83462-s002.docx]

#### COREQ 32-Item Checklist

| **Section/Topic** | **Item No.** | **Guide Questions/Description** | **Remarks** |
| --- | --- | --- | --- |
| **Domain 1: Research Team and Reﬂexivity** | | | |
| *Personal characteristics* | | | |
| Interviewer/facilitator | 1 | Which author/s conducted the interview or focus group? | Rima Nurlianti (RN) and  Christine Gracia Pratama (CGP). |
| Credentials | 2 | What were the researcher’s credentials? E.g. PhD, MD | RN is pursuing an MSc.  CGP is undertaking a PhD. |
| Occupation | 3 | What was their occupation at the time of the study? | RN is a student.  CGP is a researcher and PhD student. |
| Gender | 4 | Was the researcher male or female? | RN and CGP are female. |
| Experience and training | 5 | What experience or training did the researcher have? | RN was a system and data analyst.  CGP is a market analyst.  Both had experience in conducting qualitative interviews. |
| *Relationship with participants* | | | |
| Relationship established | 6 | Was a relationship established prior to study commencement? | No prior relationship was established with participants before the study. |
| Participant knowledge of the interviewer | 7 | What did the participants know about the researcher? e.g. personal goals, reasons for doing the research | Participants were informed about the study's purpose, research objectives, and the researcher's background before the interview. |
| Interviewer characteristics | 8 | What characteristics were reported about the interviewer/facilitator? e.g. Bias, assumptions, reasons, and interests in the research topic | The participants were aware that the researcher had survived an autoimmune condition and was a master's student with a keen interest in the field of healthcare. |
| **Domain 2: Study design** | | | |
| *Theoretical framework* | | | |
| Methodological orientation and theory | 9 | What methodological orientation was stated to underpin the study? e.g. grounded theory, discourse analysis, ethnography, phenomenology, content analysis | Thematic analysis |
| *Participant selection* | | | |
| Sampling | 10 | How were participants selected? e.g. purposive, convenience, consecutive, snowball | Purposive sampling was used to ensure diverse experiences, geographical representation, and perspectives in healthcare. |
| Method of approach | 11 | How were participants approached? e.g. face-to-face, telephone, mail, email | Participants were approached through email, social media (Instagram, LinkedIn, ResearchGate), and WhatsApp groups. |
| Sample size | 12 | How many participants were in the study? | 15 physicians and 9 patients were interviewed. |
| Non-participation | 13 | How many people refused to participate or dropped out? Reasons? | Out of 35 contacted physicians, 20 declined or did not respond. For patients, 6 out of 15 contacted individuals did not participate. |
| *Setting* | | | |
| Setting of data collection | 14 | Where was the data collected? e.g. home, clinic, workplace | Interviews were conducted via Zoom or WhatsApp video calls. |
| Presence of non-participants | 15 | Was anyone else present besides the participants and researchers? | In most cases, only the interviewer and interviewee were present during the interviews. However, in two sessions, the founder of an autoimmune community joined as a passive non-participant, assisting in reaching out to potential respondents. |
| Description of sample | 16 | What are the important characteristics of the sample? e.g. demographic data, date | Participants included physicians from various across Indonesia and patients diagnosed with autoimmune disease. |
| *Data collection* | | | |
| Interview guide | 17 | Were questions, prompts, guides provided by the authors? Was it pilot tested? | A semi-structured interview guide was developed based on prior literature and refined through expert consultations (Appendix II for physicians, Appendix IV for patients). |
| Repeat interviews | 18 | Were repeat inter views carried out? If yes, how many? | No repeat interviews were conducted. |
| Audio/visual recording | 19 | Did the research use audio or visual recording to collect the data? | Interviews were recorded with participant consent. |
| Field notes | 20 | Were ﬁeld notes made during and/or after the interview or focus group? | Field notes were taken to supplement recorded data. |
| Duration | 21 | What was the duration of the interviews or focus group? | The interview ranged from 25 to 63 minutes for physicians, with an average of 45 minutes, and from 26 to 70 minutes for patients, averaging 54 minutes. |
| Data saturation | 22 | Was data saturation discussed? | Data collection continued until thematic saturation was reached. |
| Transcripts returned | 23 | Were transcripts returned to participants for comment and/or correction? | Transcripts were not returned to participants for verification. |
| **Domain 3: Analysis and Findings** | | | |
| *Data analysis* | | | |
| Number of data coders | 24 | How many data coders coded the data? | Coding was performed by RN as a single researcher. Selected interviews were co-codes by CGP. |
| Description of the coding tree | 25 | Did authors provide a description of the coding tree? | The deductive coding approach was used to develop the coding tree. |
| Derivation of themes | 26 | Were themes identiﬁed in advance or derived from the data? | Noted in 3.4, themes were derived both inductively from participant responses and deductively based on the research questions. |
| Software | 27 | What software, if applicable, was used to manage the data? | The data were transcribed verbatim using Zoom or TurboScribe transcription features, then translated with the help of ChatGPT 4.0. MAXQDA was employed for coding and thematic analysis while Microsoft Excel was used to analyze cost-related factors. |
| Participant checking | 28 | Did participants provide feedback on the ﬁndings? | Transcripts were not returned to participants for feedback on findings. |
| *Reporting* | | | |
| Quotations presented | 29 | Were participant quotations presented to illustrate the themes/ﬁndings? Was each quotation identiﬁed? e.g. participant number | Direct quotations from participants are included in the result to illustrate key findings. |
| Data and ﬁndings consistent | 30 | Was there consistency between the data presented and the ﬁndings? | Yes, the study findings were reported clearly and consistently to accurately reflect the data collected. |
| Clarity of major themes | 31 | Were major themes clearly presented in the ﬁndings? | Yes, major themes are clearly presented in the result and discussion. |
| Clarity of minor themes | 32 | Is there a description of diverse cases or discussion of minor themes? | Yes, sub-themes are also presented with supporting evidence where relevant. |
